# Factors Associated With Intention to Adopt mHealth Apps Among Dementia Caregivers With a Chronic Condition: Cross-sectional, Correlational Study

**DOI:** 10.2196/27926

**Published:** 2021-08-31

**Authors:** Kyra Jennifer Waligora Mendez, Chakra Budhathoki, Alain Bernard Labrique, Tatiana Sadak, Elizabeth K Tanner, Hae Ra Han

**Affiliations:** 1 School of Nursing Johns Hopkins University Baltimore, MD United States; 2 Johns Hopkins Bloomberg School of Public Health Johns Hopkins University Baltimore, MD United States; 3 School of Nursing University of Washington Seattle, WA United States

**Keywords:** mHealth applications, mobile health, intention to adopt mHealth applications, dementia caregivers, family caregiving, chronic disease self-management, mobile phone

## Abstract

**Background:**

In the United States, nearly 80% of family caregivers of people with dementia have at least one chronic condition. Dementia caregivers experience high stress and burden that adversely affect their health and self-management. mHealth apps can improve health and self-management among dementia caregivers with a chronic condition. However, mHealth app adoption by dementia caregivers is low, and reasons for this are not well understood.

**Objective:**

The purpose of this study is to explore factors associated with dementia caregivers’ intention to adopt mHealth apps for chronic disease self-management.

**Methods:**

We conducted a cross-sectional, correlational study and recruited a convenience sample of dementia caregivers. We created a survey using validated instruments and collected data through computer-assisted telephone interviews and web-based surveys. Before the COVID-19 pandemic, we recruited dementia caregivers through community-based strategies, such as attending community events. After nationwide closures due to the pandemic, the team focused on web-based recruitment. Multiple logistic regression analyses were used to test the relationships between the independent and dependent variables.

**Results:**

Our sample of 117 caregivers had an average age of 53 (SD 17.4) years, 16 (SD 3.3) years of education, and 4 (SD 2.5) chronic conditions. The caregivers were predominantly women (92/117, 78.6%) and minorities (63/117, 53.8%), experienced some to extreme income difficulties (64/117, 54.7%), and were the child or child-in-law (53/117, 45.3%) of the person with dementia. In logistic regression models adjusting for the control variables, caregiver burden (odds ratio [OR] 1.3, 95% CI 0.57-2.8; *P*=.57), time spent caregiving per week (OR 1.7, 95% CI 0.77-3.9; *P*=.18), and burden of chronic disease and treatment (OR 2.3, 95% CI 0.91-5.7; *P*=.08) were not significantly associated with the intention to adopt mHealth apps. In the final multiple logistic regression model, only perceived usefulness (OR 23, 95% CI 5.6-97; *P*<.001) and the interaction term for caregivers’ education and burden of chronic disease and treatment (OR 31, 95% CI 2.2-430; *P*=.01) were significantly associated with their intention to adopt mHealth apps. Perceived ease of use (OR 2.4, 95% CI 0.67-8.7; *P*=.18) and social influence (OR 1.8, 95% CI 0.58-5.7; *P*=.31) were not significantly associated with the intention to adopt mHealth apps.

**Conclusions:**

When designing mHealth app interventions for dementia caregivers with a chronic condition, it is important to consider caregivers’ perceptions about how well mHealth apps can help their self-management and which app features would be most useful for self-management. Caregiving factors may not be relevant to caregivers’ intention to adopt mHealth apps. This is promising because mHealth strategies may overcome barriers to caregivers’ self-management. Future research should investigate reasons why caregivers with a low education level and low burden of chronic disease and treatment have significantly lower intention to adopt mHealth apps for self-management.

## Introduction

### Background

In the United States, more than 11 million family caregivers provide care to a loved one with Alzheimer disease or related dementias [1,2]. Up to 80% of the caregivers have chronic health conditions [3,4]. However, because of the high demands of caregiving responsibilities, caregivers experience challenges with their own self-management [5]. Self-management is an individual’s ability to manage or cope with the physical, psychosocial, and cultural effects of living with a chronic health condition [6].

Previous research supports that family caregivers of people with dementia perform less self-management than noncaregivers and experience worse health and well-being outcomes [7-10]. High caregiver burden and stress are barriers to self-management for family caregivers of people with dementia [5,11,12]. The COVID-19 pandemic has further exacerbated challenges to caregivers’ self-management, with preliminary research reporting that the pandemic has increased anxiety and strain among family caregivers [13,14]. In addition, family caregivers are experiencing poorer mental and physical health outcomes than noncaregivers during the COVID-19 pandemic [15]. This further highlights the critical need for innovative methods that are readily accessible and improve caregiver self-management, health, and well-being.

### Literature Review

mHealth strategies are effective in improving self-management and health outcomes of persons living with diabetes, mental health conditions, and cancer, among other chronic conditions [16-19]. However, family caregivers are less likely to use mobile apps for health-related needs than the general population [20], and fewer than 50% of the dementia caregivers use mHealth apps for their own health [21]. The reasons for these findings are largely unknown and require additional study [20,21].

The Technology Acceptance Model (TAM) is a well-known theoretical framework for exploring the factors associated with mHealth app adoption. The TAM was originally developed to explain the intention to adopt software systems [22] but has since been adapted to explore mHealth app adoption [23,24]. The TAM posits 2 technological factors that predict intention to adopt technology are perceived usefulness (beliefs about how well mHealth apps will help oneself to perform self-management) and perceived ease of using technology (one’s beliefs that using mHealth apps will take little effort) [24-26]. Perceived usefulness and perceived ease of use have been positively associated with the intention to adopt mHealth solutions among persons with a chronic condition [23,24,27] and with dementia caregivers’ intention to adopt wearable devices to manage persons with dementia [28] and caregiving-supportive technologies [29].

Prior studies have expanded the TAM to improve its utility and predictive power [23,24,28,30-32]. For example, in noncaregiver populations living with a chronic condition, social influence (perceptions that people who are important in your life believe that you should use technology) and perceptions of chronic disease threats have been associated with the intention to adopt mHealth solutions such as apps [23,24,30,31]. Perceptions that caregiving mHealth apps can prevent threats to the care recipient’s health have also been associated with the intention to adopt mHealth apps among caregivers [32]. Nevertheless, it is unclear how caregivers’ own chronic disease threats or burden may influence their intention to adopt self-management mHealth apps.

Furthermore, the findings from other studies suggest that caregiving factors may be relevant to caregivers’ intention to adopt mHealth apps. For example, in the context of mHealth apps that support caregiving, caregivers with higher caregiver burden and strain had higher mHealth app use [33], and mHealth app use reduced caregiver strain and depression [34]. However, it is unclear if these caregiving factors are relevant to caregivers’ use of mHealth apps for self-management. As caregiver burden and hours providing care per week are barriers to caregivers’ self-management [5,12], it is important to further explore how these caregiving factors may affect caregivers’ use of mHealth apps for their self-management.

In addition, racial and ethnic groups have similar rates of smartphone ownership according to national surveys [35,36], with Hispanic and Asian households having slightly higher smartphone ownership [36]; however, there are differences in whether they have downloaded an mHealth app [37,38]. Other studies have supported the existence of income and education differences in mobile device use [37,38], but that education may be a more comprehensive predictor of electronic health use than income [38]. Thus, it is also important to explore how the factors associated with mHealth adoption may differ by race or ethnicity and education to address disparities in mHealth app adoption.

### Objectives

Taken together, although much progress has been made in expanding the TAM, there is still limited knowledge of the factors associated with mHealth app adoption among dementia caregivers with a chronic condition. Caregivers are often burdened to care for their own chronic health conditions, in addition to the multimorbidities of the person with dementia, and therefore have unique barriers to self-management compared with other populations [5]. To our knowledge, there are no prior studies that have investigated the factors associated with the intention to adopt mHealth apps for self-management among caregivers living with a chronic health condition. To fill this gap, the purpose of this study is to understand factors related to the intention of family caregivers of people with dementia to adopt mHealth apps for their own chronic disease self-management. The study aims are as follows:

- Aim 1: to examine the relationships among dementia caregivers’ technological, self-management, and caregiving factors and their intention to adopt mHealth apps for self-management. Hypothesis 1: we hypothesized that technological and self-management factors would be positively, and caregiving factors would be negatively, associated with the intention to adopt mHealth apps for chronic disease self-management, controlling for the caregivers’ multimorbidities, age, gender, and income.
- Aim 2: to explore whether the caregivers’ race or ethnicity and education moderate the relationship between the study variables and caregivers’ intention to adopt mHealth apps for chronic disease self-management.

## Methods

### Study Design and Sample

We conducted a cross-sectional, correlational study and collected data in English and Spanish using computer-assisted telephone interviews and a web-based survey, both of which used the same web-based REDCap (Research Electronic Data Capture [39]) survey. Individuals were eligible for the study if they met the following criteria: aged 18 years or older; caring for a family member or friend with Alzheimer disease or related dementias; living with a chronic health condition; able to speak and understand English or Spanish; and owns, or has access to, a mobile device. Family caregivers were excluded if they, or the persons with dementia being given care, were institutionalized.

Using G*Power version 3.1.9.2 (Heinrich Heine University) and effect sizes from a recent study [23], we estimated that a sample size of 110 was needed for 85% power to detect a medium effect size with α=.05 for 2-sided tests. We also aimed to oversample minority caregivers by stratifying study recruitment. We doubled the population-based proportions of each racial or ethnic group [1] and planned to recruit 30 Black or African American, 25 Hispanic or Latino, and 11 Asian caregivers.

### Procedures

All study procedures were approved by the Johns Hopkins Medicine Institutional Review Board (IRB). The study survey was created and piloted with content experts. After entering it into REDCap, it was piloted on the web and over the phone with community members to ensure that the skip patterns, survey flow, and instructions were appropriate before implementation. As part of the survey, the team provided pictures of an evidence-based self-management mHealth app for persons with diabetes to standardize the caregivers’ conception of an mHealth app [40,41].

Data were collected in English from June 2019 to August 2020 and in Spanish from July 2020 to August 2020 (see Multimedia Appendix 1 for CHERRIES [Checklist for Reporting Results of Internet E-Surveys] checklist [42]). We recruited a convenience sample using community- and web-based methods [43]. Before the COVID-19 pandemic, the recruitment efforts focused on the Baltimore-Washington metropolitan area. After the nationwide lockdowns in March 2020, the team concentrated on web-based recruitment strategies. The study team members contacted local support groups, attended community-based events, received referrals from an Alzheimer disease treatment center and research center, and placed local newspaper advertisements. We also registered the study on the web with the Alzheimer’s Association TrialMatch service and the Clinical Trials Finder of the National Institute on Aging. These methods required people to contact the study team, be referred, or sign up to be contacted to participate. After receiving the referrals or contacts of interested people, a team member screened them for eligibility and completed a phone interview or sent a personalized link to the web-based survey, which could only be completed once.

In addition, the team recruited on the web by posting advertisements on a Johns Hopkins University online news center and on social media (Google, Facebook, and YouTube) and by sending recruitment emails through a web-based research registry (ResearchMatch). These methods included an anonymous link to the eligibility screening survey. Interested individuals could click the link, complete the eligibility survey, and begin the web-based survey, if eligible. All eligible participants received information on the study purpose, procedures, risks, and benefits and consented to participate through IRB-approved oral or web-based consent. Data were stored in the REDCap database, to which only authorized, IRB-approved team members with password-protected accounts had access. All participants who completed the study survey were remunerated with a US $10 gift card.

### Study Variables and Instruments

**Figure 1:**
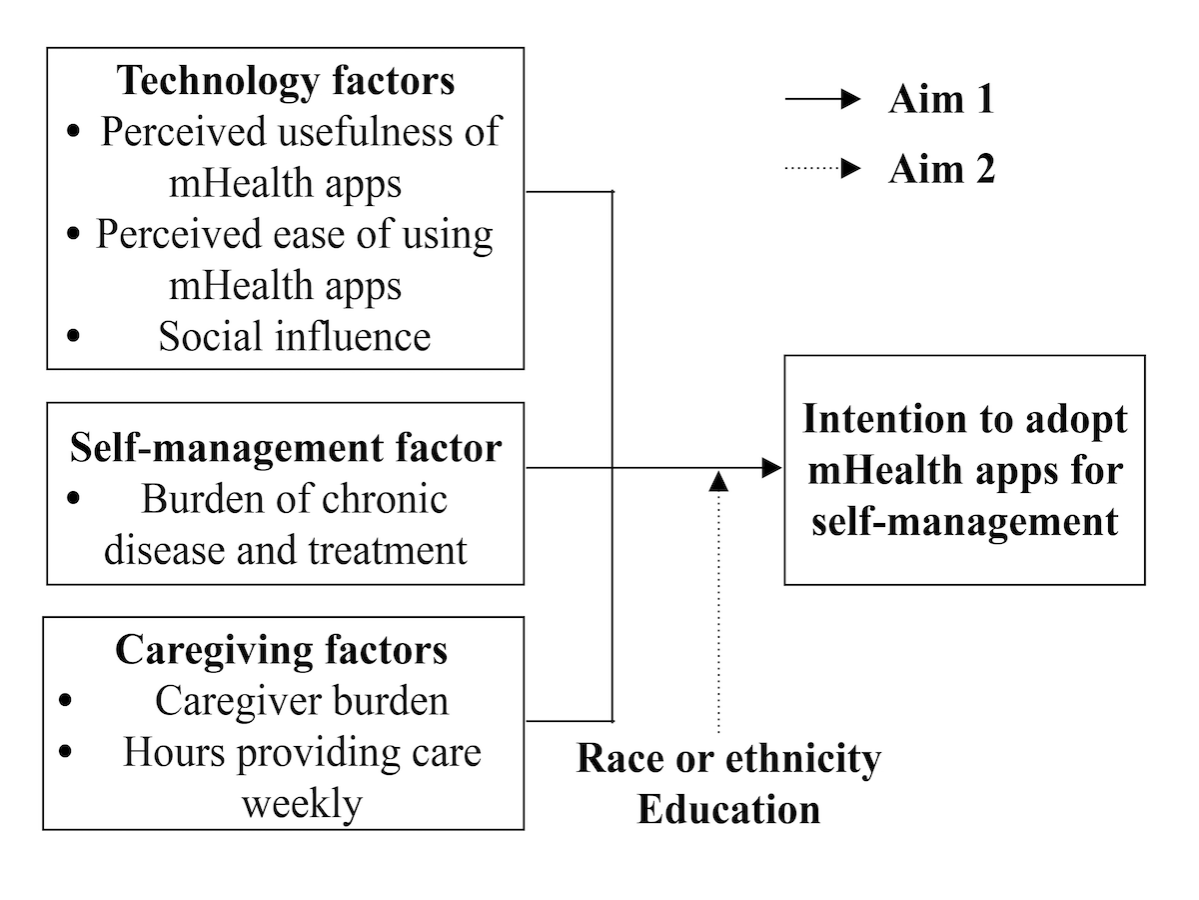
Revised Technology Acceptance Model guiding the study.

The theoretical framework guiding the study was an expanded TAM, which included the factors relevant to caregivers and their self-management [5,24,33,34]. The theoretical framework included the technology-related factors from the original and expanded TAM, caregiving factors, and a self-management factor (burden of chronic disease and treatment, defined as how much the caregivers’ chronic condition and its treatment impacts daily life). It also proposed that education and race or ethnicity moderate the relationships between technology, self-management, and caregiving factors and the intention to adopt mHealth apps (Figure 1).

To measure the sociodemographic variables, we used questions from the US Census and national surveys. Income was captured with a well-validated question of financial strain (“How hard is it for you to pay for the very basics like food, housing, medical care, and heating?”) [44]. Multimorbidity was operationalized with chronic disease counts, a list of 24 chronic conditions obtained from the Centers for Medicare & Medicaid Services Chronic Condition Warehouse [45]. Using chronic disease counts is a common method to measure multimorbidity and is significantly related to many health outcomes [46].

We operationalized the independent variables (perceived usefulness and perceived ease of use) and dependent variable (intention to adopt) using adapted versions of the 3 original TAM scales [22,23,30,47], which all had good reliability (Cronbach α>.9) and validity [26,30,47]. Researchers have modified the original scales to measure the internet and mHealth apps and reported that the modified scales had good internal consistency (Cronbach α>.8) [23,48]. For this study, we changed the original wording from “[information] system” to “mHealth app” and “in my job” to “manage my chronic condition,” as one’s job is conceptualized as self-management [22,23]. In our sample, the intention to adopt (Cronbach α=.91), perceived usefulness (Cronbach α=.96), and perceived ease of use (Cronbach α=.91) scales all had high internal consistency.

Social influence was measured using the Social Influence Scale developed when the TAM was expanded [30]. The original scale had good reliability and validity [30], and an adapted version measuring social influence in the context of the intention to adopt mHealth apps among patients with heart failure had good internal consistency (Cronbach α=.91) [23]. In our sample, the scale had good internal consistency (Cronbach α=.78).

We measured caregiver burden using the 12-item short-form version of the Zarit Burden Interview (ZBI), which has been widely used in dementia caregiving research and found to have good internal consistency, test-retest reliability, and strong correlations with the full ZBI [49-51]. In this study sample, the ZBI instrument had Cronbach α=.90. The number of hours providing care weekly was operationalized using items from the National Long-Term Care Survey that Gitlin et al [52] shortened and adapted for use with dementia caregivers. These items ask how much time caregivers spend helping a person with dementia to perform certain activities of daily living or instrumental activities of daily living. The instrument in our sample had good internal consistency (Cronbach α=.84).

Finally, caregivers’ burden of chronic disease and treatment was operationalized using the Illness Intrusiveness Ratings Scale (IIRS). This 13-item instrument measures the degree to which a disease and its treatment disrupt one’s life and activities [53]. Numerous studies have validated the IIRS in various populations with a chronic disease and have supported its reliability and validity [53]. In our caregiving sample, the IIRS had excellent internal consistency (Cronbach α=.93).

### Handling Fraudulent and Missing Data

Some web-based surveys were anonymous. Thus, fake or fraudulent survey responses were potential issues that could affect research integrity [54]. REDCap does not collect IP addresses or cookies. Thus, we included other methods for detecting and handling fraudulent responses. For example, we reviewed the web-based survey completion times, response patterns, participants’ contact information, and contact attempts. Furthermore, the participants needed to fill out a petty cash voucher to be reimbursed for the study, which allowed the team to verify information for some respondents; however, not all participants included in the analyses completed a voucher. Guided by the recommendations in the study by Teitcher et al [54], we excluded survey responses (14/186, 7.5%) that had (1) very short survey completion times (limits established by mock survey and average completion times), (2) unvalidated email addresses (eg, no responses to emails), and (3) inconsistent response patterns (eg, *Christmas tree* answers).

Next, we examined the data for missing, *don’t know*, and *refused to answer* values. All variables had less than 4% *don’t know* and 1% missing values, except for the question asking participants if they had other chronic conditions (5/117, 4.3% missing). We treated *don’t know* and *refused to answer* choices as missing values and imputed a neutral or very conservative (eg, no chronic condition) value for each missing answer.

### Data Analyses

#### Aim 1: Testing Hypothesis 1

We used descriptive statistics (mean, median, and SD) to summarize the variables and examined the distributions of independent and dependent continuous variables. We also examined the correlation matrix of bivariate associations between the independent variables and the dependent variable. All TAM variables had left-skewed distributions, with 70.1% (82/117) of the participants choosing values above neutral (somewhat agree and higher; Table S1 in Multimedia Appendix 2). We originally planned to model the outcome using linear regression; however, the data violated the assumptions of linear regression (the predicted values were associated with residual values) even after linear transformations of the outcome. Thus, we dichotomized the outcome and modeled it using multiple logistic regression.

We applied a data-driven and theoretical approach to dichotomize the TAM variables into high and low groups. Specifically, we used an approximate median split (55/45) and theoretical cutoff points for people who moderately agreed to strongly agreed that they intended to adopt mHealth apps and perceived mHealth apps as useful and easy to use. We used a similar approach for the social influence variable (people who more than somewhat agreed). The self-management and caregiver burden variables were normally distributed; thus, we dichotomized these variables at their medians. Finally, caregiving time was dichotomized into high (≥21 hours/week) and low (<21 hours/week), following a published cutoff score [55].

For hypothesis testing, each independent variable was individually regressed onto the outcome, controlling for age, gender, income, and multimorbidity, which have been associated with technology adoption in prior studies [30,56,57]. Next, any independent variables in the initial adjusted regression models with *P*<.15 were included in the final regression model [58]. We also assessed for multicollinearity in the final model, but statistics supported that multicollinearity was not an issue (average variance inflation factor=1.45).

#### Aim 2: Exploring Moderation

For moderation testing, we used the final model from the aim 1 analyses. Subsequently, we dichotomized race or ethnicity into White, non-Hispanic and people of color and education at its median (16 years). We created interaction terms for each dichotomized independent variable: race or ethnicity and education. All statistically significant interaction terms (*P<.*05) were included in the final model for aim 2.

## Results

### Sample Characteristics

**Figure 2:**
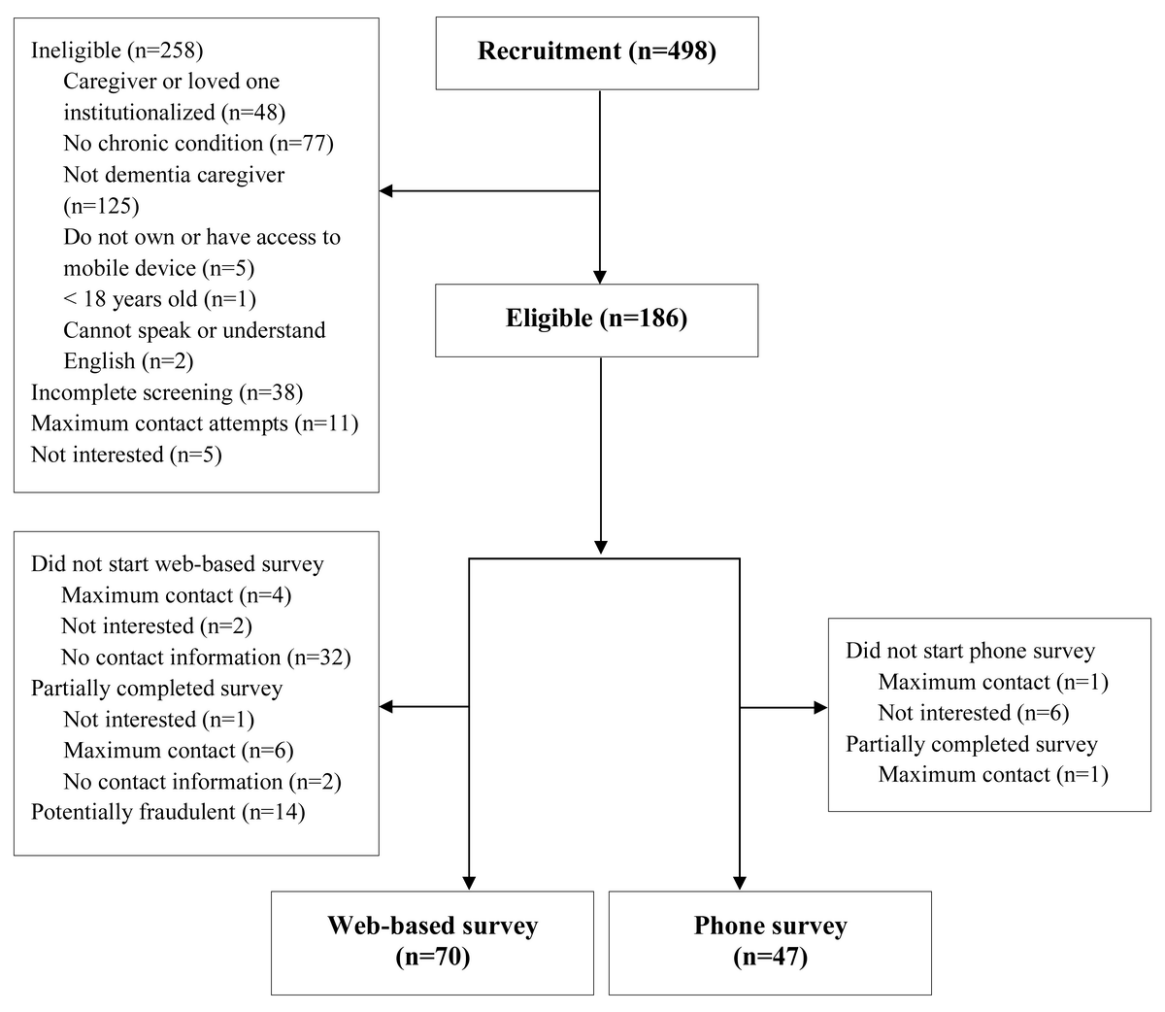
CONSORT (Consolidated Standards of Reporting Trials) flow chart of study recruitment.

The study team recruited 498 people interested in the study (Figure 2). The final sample consisted of 117 eligible caregivers; 59.8% (70/117) completed the web-based survey, and 40.1% (47/117) completed the phone survey (Table 1). Only 1 Spanish-speaking caregiver completed the Spanish web-based survey, although 79 were recruited and 11 were eligible.

**Table 1:** Sociodemographic characteristics of the study sample of dementia caregivers living with a chronic health condition (N=117).

Sociodemographic characteristic				Values
Age (years), mean (SD)				52.7 (17.4)
Education (years), mean (SD)				16 (3.3)
**Gender, n (%)**				
	Female			92 (78.6)
	Male			25 (21.4)
**Race or ethnicity, n (%)**				
	White or non-Hispanic			54 (46.2)
	Black or African American			31 (26.5)
	Hispanic or Latino			17 (14.5)
	Asian			11 (9.4)
	Native American			2 (1.7)
	Multiple			2 (1.7)
**Marital status, n (%)**				
	Married or living as married			58 (49.6)
	Never married			34 (29.1)
	Widowed, divorced, or separated			23 (219.7)
	Refused to answer			2 (1.7)
**Income (financial strain), n (%)**				
	Not at all or not very difficult			53 (45.3)
	Somewhat difficult			48 (41)
	Very or extremely difficult			16 (13.7)
**Chronic health conditions^a^**				
	Value, mean (SD)			4 (2.5)
	**Common chronic conditions, n (%)**			
		Hypertension	55 (47)	
		Depression	50 (42.7)	
		Hyperlipidemia	40 (34.2)	
		Rheumatoid arthritis or osteoarthritis	39 (33.3)	
		Asthma	32 (27.4)	
		Migraine or chronic headache	32 (27.4)	
		Mental health condition	27 (23.1)	
		Diabetes (type 1 and 2)	24 (20.5)	
		Cataracts	23 (19.7)	
**Relationship to person with dementia, n (%)**				
	Child or child-in-law			53 (45.3)
	Grandchild			22 (18.8)
	Spouse or significant other			22 (18.8)
	Other (family member)			12 (10.3)
	Friend			8 (6.8)
**Paid to provide care, n (%)**				
	No			106 (90.6)
	Yes			10 (8.5)
	Refused to answer			1 (0.9)
Time spent caregiving per week (hours), mean (SD)				27.3 (30.8)

^a^Nine most common chronic conditions in the sample.

On average, the caregivers were aged approximately 53 years (SD 17.4), with an age range of 19-88 years. Most of the sample consisted of women (92/117, 78.6%), and more than half were minorities (63/117, 53.8%). Approximately half of the caregivers were married or living as married (58/117, 49.6%), and 29.1% (34/117) had never married. On average, the caregivers had completed 16 (SD 3.3) years of education, and more than half of the caregivers (64/117, 54.7%) reported that it was somewhat difficult to extremely difficult to manage on their income. Of the 117 participants, 53 (45.3%) were the child or child-in-law of the person with dementia, with an even proportion of caregivers being the spouse or significant other (22/117, 18.8%) or grandchild (22/117, 18.8%). The caregivers had, on average, 4 (SD 2.5) chronic health conditions, with a range of 1-15 (Table 1).

The web-based survey respondents were, on average, approximately 20 years younger (t_115_=–7.81; *P*<.001), had one less chronic condition (t_115_=–3.15; *P*=.002), provided 25 fewer hours of care per week (t_53_=–4.07; *P*<.001), and had a 15-point higher burden of chronic disease and treatment (t_115_=4.08; *P*<.001). In addition, a greater proportion of the web-based survey respondents were the grandchild of the person with dementia (22/70, 31% compared with 0/47, 0%; *χ*^2^_4_=20.5; *P*<.001). There were no other significant differences in the sociodemographic characteristics or main variables between the web-based and phone survey respondents.

### Aim 1 Results: Testing Hypothesis 1

In bivariate associations, the intention to adopt mHealth apps was significantly associated with perceived usefulness (*χ*^2^_1_=49.8; *P*<.001), perceived ease of use (*χ*^2^_1_=28.7; *P*<.001), and social influence (*χ*^2^_1_=10.2; *P*=.002). Furthermore, perceived usefulness explained 52% of the variance in the outcome (Nagelkerke *R^2^*=0.52). However, the caregivers’ intention to adopt mHealth apps was not significantly associated with burden of chronic disease and treatment (*χ*^2^_1_=3.2; *P*=.09), caregiver burden (*χ*^2^_1_=0.6; *P*=.45), or hours spent caregiving per week (*χ*^2^_1_=1.8; *P*=.18).

After controlling for age, gender, income, and multimorbidity, we found that perceived usefulness (odds ratio [OR] 31, 95% CI 10-94; *P*<.001), perceived ease of use (OR 10.2, 95% CI 4.1-25; *P*<.001), social influence (OR 3.5, 95% CI 1.6-7.7; *P*=.002), and burden of chronic disease or treatment (OR 2.3, 95% CI 0.91-5.7; *P*=.08) were individually associated with the caregivers’ intention to adopt mHealth apps with a *P*<.15, the a priori screening criteria. Caregiver burden (OR 1.3, 95% CI 0.57-2.8; *P*=.57) and hours spent caregiving per week (OR 1.7, 95% CI 0.77-3.9; *P*=.18) were not associated with the intention to adopt mHealth apps in adjusted models with *P*<.15, our a priori screening criteria, and were not included in the final aim 1 model.

The final aim 1 model is presented in Table 2. After controlling for other independent variables, only perceived usefulness was statistically significantly associated with the intention to adopt mHealth apps (OR 15, 95% CI 4.3-51; *P*<.001), although the overall model was significant (Hosmer-Lemeshow test, *χ*^2^_8_=11.5; *P*=.18). Specifically, caregivers who had high perceptions that mHealth apps were useful to their self-management had 15 higher odds of intending to adopt mHealth apps compared with those with low perceptions of mHealth apps being useful for self-management.

**Table 2:** Final multiple logistic regression models for aims 1 and 2.

Variable^a^		Adjusted odds ratio (95% CI)	
		Aim 1 final model^b^	Aim 2 final model^c^
**Step 1: control variables**			
	Multimorbidity	0.87 (0.7-1.1)	0.93 (0.74-1.2)
	Age (years)	1.03 (0.99-1.1)	1.03 (0.99-1.1)
	Gender	0.6 (0.16-2.2)	0.41 (0.1-1.6)
	Income	0.66 (0.21-2.1)	0.63 (0.16-2.4)
**Step 2: independent variables**			
	Perceived usefulness	15 (4.3-51)^d^	23 (5.6-97)^d^
	Perceived ease of use	3.1 (0.95-10)	2.4 (0.67-8.7)
	Social influence	1.9 (0.64-5.5)	1.8 (0.58-5.7)
	Burden of chronic disease or treatment (IIRS^e^)	2.5 (0.68-9.2)	0.31 (0.038-2.5)
**Step 3: interaction term**			
	Education	—^f^	0.24 (0.034-1.6)
	IIRS × education	—	31 (2.2-430)^g^

^a^Measurement of variables is as follows (variable: measurement)—multimorbidity: chronic disease counts from the Centers for Medicare & Medicaid Services Chronic Condition Warehouse [45]; age, gender, education: questions from the US Census and other national surveys; income: Likert-type question asking, “How hard is it for you to pay for the very basics like food, housing, medical care, and heating?” [44]; perceived usefulness: Perceived Usefulness Scale modified for mHealth apps [22,23]; perceived ease of use: Perceived Ease of Use Scale modified for mHealth apps [22,23]; social influence: Social Influence Scale modified for mHealth apps [23,30]; burden of chronic disease or treatment: Illness Intrusiveness Ratings Scale [53].

^b^Aim 1 final model statistics: Hosmer-Lemeshow test, *P*=.18; Nagelkerke *R^2^=*0.57.

^c^Aim 2 final model statistics: Hosmer-Lemeshow test, *P*=.82; Nagelkerke *R^2^=*0.62.

^d^*P*<.001.

^e^IIRS: Illness Intrusiveness Ratings Scale.

^f^Variables not tested in aim 1 analyses.

^g^*P*=.01.

### Aim 2 Results: Exploring Moderation

After exploring moderation, race or ethnicity did not significantly change the relationship between the independent variables and the outcome. The only statistically significant interaction term associated with the caregivers’ intention to adopt mHealth apps was education and burden of chronic disease or treatment (OR 31, 95% CI 2.2-430; *P*=.01; see Tables S2 and S3 in Multimedia Appendix 2 for group comparisons).

In the final model with the interaction terms included, perceived usefulness (OR 23, 95% CI 5.6-97; *P*<.001) and the interaction term for education and burden of chronic disease or treatment (OR 31, 95% CI 2.2-430; *P*=.01) were statistically significantly associated with the intention to adopt mHealth apps. Specifically, the odds of intending to adopt an mHealth app were 23 times greater among caregivers with high beliefs that mHealth apps are useful for self-management compared with those with low beliefs that mHealth apps are useful, controlling for all other variables. In addition, the odds of intending to adopt mHealth apps for self-management were 31.6 times greater among caregivers with a high level of education and high burden of chronic disease and treatment compared with those with a low level of education and low burden of chronic disease and treatment. The other independent and control variables were not significant, although the overall model was significant (Hosmer-Lemeshow test, *χ*^2^_8_=4.4; *P*=.82) and explained 62% of the variance in the outcome.

## Discussion

### Principal Findings

The aim of this study was to explore the barriers to and facilitators of the intention to adopt mHealth apps for self-management among dementia caregivers with a chronic condition. In our study of 117 caregivers, we found that perceived usefulness explained 52% of the variance and was the strongest predictor of caregivers’ intention to adopt mHealth apps for their self-management. Furthermore, after controlling for perceived usefulness, other independent variables were no longer significantly associated with the intention to adopt mHealth apps. None of the caregiving variables were significantly associated with the caregivers’ intention to adopt mHealth apps in any model. We also found that caregivers with a high education level and greater burden of chronic disease and treatment had a significantly greater intention to adopt mHealth apps for their self-management than those with a low education level and low burden of chronic disease and treatment.

Perceived usefulness has consistently been a strong predictor of the intention to adopt mHealth solutions among older adults and persons with a chronic condition [24,31,59]. In later iterations of the TAM, perceived usefulness constructs had strong power to predict and were a key determinant of the intention to adopt various health technologies, including mHealth solutions [24,31,59]. For example, Dou et al [24] reported that perceived usefulness had a strong, significant positive association with the intention to adopt mHealth apps, whereas perceived ease of use was only significant when the mHealth apps were also perceived as useful. When designing mHealth app interventions for older adults with a chronic condition, it is critical to apply a user-centered design approach, which includes understanding which app features are most relevant for certain populations [60]. Future research should consider the specific features of mHealth apps that caregivers perceive as useful to their self-management, which can further facilitate their mHealth app adoption.

Although only perceived usefulness was statistically significant, perceived ease of use was clinically meaningful because caregivers who believed that mHealth apps were easy to use had 2.4 times greater intention to adopt them. This finding may not have reached statistical significance because of insufficient sample size or the age of our caregiving sample, reflecting a younger, more tech-savvy generation. For example, we only included caregivers who owned or had access to mobile devices. Previous studies have found that younger adults have higher mobile device ownership and mobile app use, as well as better technology skills than older generations [57,61]. In a larger sample of 381 dementia caregivers who were older adults (mean age 63 years, SD 13 years), Xiong et al [62] reported that ease of installation and use of caregiving-supportive technologies were the most important factors in the caregivers’ decision to adopt these technologies, although the study did not investigate perceived usefulness. Other research has supported the finding that the ease of using mHealth apps is an important consideration for older adults because of cognitive, motivational, and physical barriers [60,63,64]. Nevertheless, the study by Burstein et al [29] found that after controlling for perceived usefulness, ease of use was not significantly associated with willingness to adopt caregiving-supportive technologies among older adults (mean age 59 years), similar to our findings. Taken as a whole, existing TAM research suggests that perceived usefulness is a significant facilitator of caregivers’ intention to adopt technology, although ease of use may be more salient for older adult caregivers or for sustained engagement with mHealth apps, rather than for adoption [27].

In our caregiving sample, social influence had a larger, although statistically nonsignificant, OR of 1.8 (95% CI 0.58-5.7). Existing studies on the significance of social influence with regard to health-related technology adoption have been mixed. Some studies report that social influence is a significant facilitator of health-related technology adoption among general consumers [65] and patients with heart failure [23], whereas others report that it is not significant among older adults [59]. Among dementia caregivers, Dai et al [28] found that social influence significantly positively predicted the caregivers’ intention to adopt wearable devices to manage the care recipient’s health. However, social influence was not significantly associated with mHealth app adoption for caregivers’ self-management in our sample.

This discrepancy in the findings may be related to differences in population, type of technology, or sample demographics. For example, our sample consisted of caregivers who were predominantly English-speaking, middle-aged, and the child or grandchild of the person with dementia. Compared with our caregiving sample, the sample in the study by Dai et al [28] consisted of younger caregivers, with more men, who lived in sub-Saharan Africa. In addition, our outcome investigated mHealth apps for caregivers’ self-management, which is different from caregiving technologies [28]. Caregivers have a high interest in adopting caregiving technologies, but much less is known about their interest in adopting technologies for their self-management [32,66]. In-depth qualitative investigations can improve our understanding of mechanisms by which social influence impacts technology adoption; and whether social influence is more important for certain groups (eg, older caregivers) or technologies (caregiving vs self-management technologies).

In our study, social influence reflected subjective norm (perceptions that people who are important in your life believe that you should perform an action) from the Theory of Reasoned Action [23,30]. However, social support is another construct relevant to older adults’ technology adoption that reflects the quality of social relationships [67,68] and may affect caregivers’ adoption of mHealth apps. For example, previous qualitative studies have suggested that some dementia caregivers have poor technology literacy and rely on family to assist with using technology [68,69], although quantitative studies have found that dementia caregivers have good eHealth literacy [21]. A recent cross-sectional study supported that both subjective norm and social relationships were significant correlates of the intention to adopt mHealth apps among older adults [70]. Thus, future research is warranted to understand how social support and social influence may interact to affect mHealth app adoption among caregivers and how social support or influence may differ according to caregivers’ technology literacy.

Furthermore, our sample size (n=117) was smaller than the samples in the studies by Dai et al (n=350) [28], Cajita et al (n=129) [23], and Kim and Park (n=728) [65]. Similar to the ease-of-use variable, it is possible that social influence has a smaller effect size, requiring larger samples to detect a significant relationship. Researchers should consider conducting meta-analyses to determine the effect sizes required to detect statistically significant relationships among the TAM variables. A meta-analysis will provide precise effect size estimates, with greater generalizability.

Caregiver burden and the hours spent caregiving did not contribute significantly to explaining the intention to adopt mHealth apps among family caregivers. Although these 2 caregiving factors negatively impact caregivers’ self-care [5], our findings suggest that they may not be relevant to caregivers’ decisions about whether to adopt mHealth apps for self-management. The median time spent caring in our sample (18.3 hours) was lower than the US population average for dementia caregivers (26.3 hours) [1], although our sample had high levels of burden (mean ZBI score of 21), which reflects the findings of other researchers [49,71,72]. Nevertheless, our sample consisted of middle-aged and well-educated caregivers. Thus, additional research is needed to test whether these findings can be extrapolated to caregivers who are older adults and less educated.

We found that the burden of chronic disease and treatment was not significantly associated with caregivers’ intention to adopt mHealth apps. Our study finding conflicts with that of existing studies. Other researchers have found that perceived disease threats were significantly associated with the intention to adopt mHealth solutions among persons with a chronic condition [24,31]. A possible explanation is that our concept and the methods we used to measure it were different. We examined the current burden of chronic disease and treatment on caregivers’ lives, not their perceptions of the future consequences of a disease, as in previous studies [24,31]. Thus, it is possible that the current burden of chronic disease and treatment may not motivate the adoption of mHealth apps compared with the future perceived threats of a chronic disease. Further research is required to explore this proposition.

In our sample, the caregivers’ education and burden of chronic disease and treatment interacted to produce a greater and significant effect on their intention to adopt mHealth apps. The OR (31, 95% CI 2.2-430) should be interpreted with caution because of the smaller number of caregivers in the high and low groups (Table S3 in Multimedia Appendix 2). To the best of our knowledge, very few studies have investigated how sociodemographic variables interact with chronic disease or self-management variables to affect technology adoption. A prior study investigated how age and perceived disease threat interacted to influence the intention to adopt an mHealth app and found that it was not statistically significant [31]. As prior studies have not yet examined how education and chronic disease factors may interact to affect the intention to adopt mHealth apps, additional research is needed to support this finding.

Interpreted in the context of existing research, our study offers new insights into the factors related to caregivers’ intention to adopt mHealth apps for self-management. However, additional research is still needed to maximize mHealth app adoption in this population. Furthermore, the diversity of populations, mHealth strategies, and study findings substantiate the importance of user-centered design and the development of mHealth solutions with the end users as key stakeholders [60,66]. Future research should involve dementia caregivers as stakeholders throughout the process of conceptualizing, designing, and testing mHealth strategies for their self-management.

### Limitations

This study has some limitations. We recruited a convenience sample using community-based (Baltimore, Maryland) and web-based methods. Thus, our results may not be generalizable to all family caregivers of people with dementia, such as those who lack access to the internet or social media. However, web-based recruitment methods enabled us to reach a larger caregiving population across the United States, which may also improve the external validity of the findings. In addition, this study was cross-sectional; thus, relationships are associative, not causal. Another limitation is that only 1 Spanish-speaking caregiver completed the survey, although 11 were eligible. We speculate that this was due to the caregivers’ difficulties with navigating the REDCap survey, which does not allow researchers to change the language of prebuilt, English-only survey buttons and functionalities. The attrition of Spanish-speaking caregivers occurred when they navigated to a different part of the survey with nonmodifiable, English-only REDCap buttons. Future researchers should consider this critical limitation of the REDCap platform.

Another limitation is that the study was originally powered for linear regression. As our data violated the assumptions of linear regression, we needed to use logistic regression. This change increased the models’ degrees of freedom and reduced the power to detect differences among groups. Post hoc power analyses indicated that our study had 80% power to detect an OR of 3 or higher to be statistically significant at α=.05. Thus, we may be making a type II error with some of the independent variables in our final model (such as perceived ease of use and social influence). However, in scatterplot matrices, we did not observe a linear relationship between the caregiving factors and the outcome, thus reinforcing our finding that the caregiving variables may not be relevant to caregivers’ intention to adopt mHealth apps.

### Conclusions

In our sample of caregivers with one or more chronic conditions, the perceived usefulness of mHealth apps was the strongest and most significant variable associated with their intention to adopt mHealth apps for self-management. Although ease of use and social influence were not statistically significant, they were clinically significant with larger ORs. Future research is needed to determine which app features are most useful for caregivers’ self-management, estimate effect sizes for sample size calculations, and systematically review how relationships vary by population or type of mHealth strategy.

Our findings also support the theory that the caregiving factors may not influence caregivers’ intention to adopt mHealth apps for self-management. Thus, mHealth solutions may overcome the barriers to caregivers’ self-management. Furthermore, caregivers with a high education level and greater burden of chronic disease and treatment have a higher likelihood of intending to adopt mHealth apps for self-management. Future research should explore the mechanisms by which education and self-management may interact.

Engaging dementia caregivers as stakeholders throughout the process of mHealth app conception, design, and testing can promote their adoption of mHealth apps. This process of user-centered design ensures that these apps are useful and easy to use, addresses factors relevant to caregivers, and builds support systems that encourage adoption.

## Appendix

Multimedia Appendix 1 CHERRIES (Checklist for Reporting Results of Internet E-Surveys) checklist.

Multimedia Appendix 2 Additional data on study variables and cross-tabulations for interaction term.

## Abbreviations

CHERRIES: Checklist for Reporting Results of Internet E-Surveys
IIRS: Illness Intrusiveness Ratings Scale
IRB: Institutional Review Board
OR: odds ratio
REDCap: Research Electronic Data Capture
TAM: Technology Acceptance Model
ZBI: Zarit Burden Interview
