# Supplementary material for: Factors Associated With Intention to Adopt mHealth Apps Among Dementia Caregivers With a Chronic Condition: Cross-sectional, Correlational Study
Source: JMIR Mhealth Uhealth. 2021 Aug 31;9(8):e27926. doi: 10.2196/27926 (PMC8441609; doi:10.2196/27926)
Supplement: Multimedia Appendix 1 [file mhealth_v9i8e27926_app1.pdf]

CHERRIES Checklist

**Design**	Describe survey design	Target population: Individuals were eligible for the study if they met the following criteria: aged 18 and older, caring for a family member or friend with Alzheimer's disease or related dementias, living with a chronic health condition, could speak and understand English or Spanish, and owned or had access to a mobile device. Family caregivers were excluded if they or the person with dementia were institutionalized.<br>We recruited a convenience sample using community-based and online methods.
**IRB (Institutional Review Board) approval and informed consent process**	IRB approval	All study procedures were approved by Johns Hopkins Medicine IRB.
	Informed consent	All eligible participants received information on study purpose, procedures, risks, and benefits and consented to participate through IRB-approved oral or online consents.
	Data protection	Data was stored in the RedCap database, where only authorized, IRB-approved team members with password-protected accounts had access.
**Development and pre-testing**	Development and testing	We developed the survey with well-validated instruments. The study survey was created and piloted with content experts, then after entry into RedCap, piloted online and over the phone with community members to ensure skip patterns, survey flow, and instructions were appropriate before implementation. We also collected data on time to complete phone and online surveys during the pilot phase.
**Recruitment process and description of the sample having access to**	Open survey versus closed survey	Both open and closed surveys were used, depending on the recruitment strategy. Recruitment methods that required people to contact the study team, be referred, or sign up to be contacted were closed surveys. If eligible, these participants
	Contact mode	
	Advertising the survey	

**the questionnaire**		completed the phone interview or were sent a personalized link to the online survey, which could only be completed once. Online recruitment that involved posting ads (e.g., online university news center, social media) and sending recruitment emails through ResearchMatch were open surveys. These methods included an anonymous link to the eligibility screening survey, where interested individuals could click the link, do the eligibility survey, and then begin the online survey if eligible.
**Survey administration**	Web/E-mail	The online survey was stored in and administered through RedCap. Open surveys create a new "record;" though closed surveys require members of the study team to create a "record" and then send a personalized survey link to participants (or orally administer the online survey to participants over the phone).
	Context	Alzheimer's Association's TrialMatch targets populations interested in Alzheimer's disease and related dementia research studies, and the National Institute of Aging's Clinical Trials Finder targets populations interested in research studies for aging populations. ResearchMatch is an online recruitment registry partially funded through an NIH grant, where people register for an account and consent to be contacted for research. The online university news center was Johns Hopkins The Hub, which targets Johns Hopkins University students, faculty, staff, and affiliates. Social media targets general audiences, not necessarily focused on research.
	Mandatory/voluntary	The survey was voluntary.
	Incentives	All participants who completed the study survey were remunerated with a $10 gift card.

	Time/Date	Data were collected in English from June 2019 to August 2020 and in Spanish from July 2020 to August 2020.
	Randomization of items or questionnaires	Randomization of items was not used.
	Adaptive questioning	Adaptive questioning was not used.
	Number of Items	The study survey had an average of 28 items per page.
	Number of screens (pages)	Eligibility screening was 1 page; the results of eligibility was another page; informed consent was 1 page; and the study survey had 3 pages.
	Completeness check	All items included a "Refused" answer choice and did not require a response.<br><br>Research team members reviewed completed surveys for missing answers. Email addresses were collected to contact participants about missing answers. If participants provided permission, we resent a link to the survey page with missing answers and provided a survey return code required to re-access the survey page (unique to each survey page and each participant).
	Review step	The survey did not allow participants to review their answers. Once the survey page was submitted, participants required a survey return code to re-access answers, which was only visible to team members (unless participants chose to save and return later).
**Response rates**	Unique site visitor	RedCap does not collect IP addresses or cookies.
	View rate (Ratio of unique survey visitors/unique site visitors)	We cannot determine how many people visited the online survey. RedCap only provides information on numbers of people who submit the first page of the survey. In this study, the first page was the eligibility screening. Thus, we only have access to the

		number of people who submitted the eligibility screening.
	Participation rate	498 interested (373 open survey/submitted online eligibility; 125 closed online or phone survey)<br>186 eligible<br>156 consented (156/498= 31.3%)
	Completion rate	156 consented<br>117 completed survey (75%)
**Preventing multiple entries from the same individual**	Cookies used	RedCap does not collect IP addresses or cookies for open surveys. Thus, we included other methods of detecting and handling fraudulent responses. For example, we reviewed online survey completion times, response patterns, participants' contact information, and contact attempts. Furthermore, participants needed to fill out a petty cash voucher to be reimbursed for the study, which allowed the team to verify information for some respondents. However, not all participants included in analyses completed a voucher. It provided another method of verifying participants. Guided by Teitcher and colleagues' recommendations [54], we excluded survey responses (n=14) that had: 1) Very short survey times (limits established by mock survey and average completion times); 2) Unvalidated emails (e.g. no responses to emails); and 3) Inconsistent response patterns (e.g. "Christmas tree" answers).
	IP check	
	Log file analysis	
	Registration	In closed surveys, each unique participant was a "record." Participants could choose to save and return later, in which they were provided a unique access code to return and complete the survey at a later time. Once each survey page was submitted, it could no longer be accessed without a code. In order to re-access a completed survey (or a page in the survey), participants would need an access code (visible to team members, but

		not participants, unless they chose to save and return later).
**Analysis**	Handling of incomplete questionnaires	Only completed questionnaires were analyzed.
	Questionnaires submitted with an atypical timestamp	We collected timestamps for each survey page and chose cut-offs accordingly:<br>Demographics <60 seconds<br>Technology acceptance model scales <45 seconds<br>Caregiving/Illness intrusiveness ratings scale <90 seconds<br>We chose these cutoffs based on pilot tests. Younger pilot testers (≤30 years) were able to complete the survey in these time limits by skimming and non-randomly selecting answers.
	Statistical correction	No statistical correction methods were used.
