# Supplementary material for: Factors Associated With Intention to Adopt mHealth Apps Among Dementia Caregivers With a Chronic Condition: Cross-sectional, Correlational Study
Source: JMIR Mhealth Uhealth. 2021 Aug 31;9(8):e27926. doi: 10.2196/27926 (PMC8441609; doi:10.2196/27926)
Supplement: Multimedia Appendix 2 [file mhealth_v9i8e27926_app2.pdf]

Multimedia Appendix 2

Table S1. Summary of descriptive statistics for independent and dependent variables.

Study Variables	Median (Range)	Mean (SD)
**Dependent variable**		
Intention to adopt mHealth applications	8 (0- 12)	7.7 (3.7)
**Independent variables**		
Perceived usefulness	19 (0- 24)	16.8 (6.8)
Perceived ease of use	23 (1- 30)	21.7 (7)
Social influence	12 (2- 18)	11.9 (4.2)
Caregiver burden	21 (0- 43)	21.3 (10.2)
Hours caregiving per week	18 (0.43- 212)	27.3 (30.8)
Burden of chronic disease/treatment	29 (0- 78)	29.7 (20.7)

Table S2. Crosstabulation of education and burden of chronic disease/treatment interaction term and outcome, intention to adopt mHealth applications (apps).

		**Intention to adopt mHealth apps**		
		0 (Low)	1 (High)	**Total**
**IIRS * Education**	0 (Low)	63	26	89
	1 (High)	11	17	28
	**Total**	74	43	117

Table S3. Group comparisons of burden of chronic disease/treatment (IIRS) and intention to adopt mHealth applications (apps) by education level.

			**Intention to adopt mHealth apps**		
			0 (Low)	1 (High)	**Total**
**Low IIRS (0-29)**	**Low education (0-15)**	Count	10	7	17
		% within Education	58.8%	41.2%	100.0%
	**High education (16 & up)**	Count	32	10	42
		% within Education	76.2%	23.8%	100.0%
	**Total**	Count	42	17	59
		% within Education	71.2%	28.8%	100.0%
**High IIRS (30-78)**	**Low education (0-15)**	Count	21	9	30
		% within Education	70.0%	30.0%	100.0%
	**High education (16 & up)**	Count	11	17	28
		% within Education	39.3%	60.7%	100.0%

	**Total**	Count	32	26	58
		% within Education	55.2%	44.8%	100.0%
**Total**	**Low education (0-15)**	Count	31	16	47
		% within Education	66.0%	34.0%	100.0%
	**High education (16 & up)**	Count	43	27	70
		% within Education	61.4%	38.6%	100.0%
	**Total**	Count	74	43	117
		% within Education	63.2%	36.8%	100.0%
